# Don’t let COVID-19 disrupt campus climate surveys of sexual harassment

**DOI:** 10.1073/pnas.2018098117

**Published:** 2020-09-18

**Authors:** Kathryn J. Holland, Lilia M. Cortina, Vicki J. Magley, Arielle L. Baker, Frazier F. Benya

**Affiliations:** ^a^Department of Psychology, University of Nebraska–Lincoln, Lincoln, NE 68588;; ^b^Women’s & Gender Studies Program, University of Nebraska–Lincoln, Lincoln, NE 68588;; ^c^Department of Psychology, University of Michigan, Ann Arbor, MI 48109;; ^d^Department of Women's and Gender Studies,University of Michigan, Ann Arbor, MI 48109;; ^e^Department of Management and Organizations, University of Michigan, Ann Arbor, MI 48109;; ^f^Department of Psychological Sciences, University of Connecticut, Storrs, CT 06269;; ^g^Policy and Global Affairs Division, National Academies of Sciences, Engineering, and Medicine, Washington, DC 20001

Surveying a campus community about sexual harassment can be a daunting task during normal times. It’s especially daunting during a pandemic. Institutional leaders may balk at committing scarce resources to survey efforts. Some may wonder how to interpret results that look dramatically different from prior assessments. Also, they may worry about adding to the burdens of already stressed staff, faculty, and students. Indeed, these concerns and complexities came up recently within the work of the National Academies’ Action Collaborative on Preventing Sexual Harassment in Higher Education (1).

This Action Collaborative grew out of the 2018 National Academies of Sciences, Engineering, and Medicine (NASEM) consensus study report on sexual harassment in academic science, engineering, and medicine (2). Over 60 academic and research institutions and key stakeholders sought to work together to identify, develop, implement, and evaluate ways of preventing and addressing sexual harassment in higher education. Action Collaboratives are a relatively new type of activity at the National Academies [others include Clinician Well-Being and Resilience (3) and Countering the US Opioid Epidemic (4)]. Building on the National Academies’ long history of convening stakeholders and gathering research to inform decision makers and the public, Action Collaboratives provide a space for organizations and individuals to exchange information, ideas, and strategies around topics of mutual interest and concern, create new and innovative solutions, and take collective action.

When COVID-19 disrupted plans for learning and work in higher education, representatives from member institutions in our Action Collaborative asked whether they should continue campus climate surveys, and if so, how they should proceed. Here we address these questions using our extensive experience with sexual harassment research and policy. Three of us are members of the Action Collaborative’s Advisory Committee (K.J.H., L.M.C., and V.J.M.) and specialize in the psychological study of sexual harassment and violence. Two of us (A.L.B. and F.F.B.) are program officers at NASEM and currently direct and manage the Action Collaborative.

We understand and appreciate the concerns institutions have about conducting campus climate surveys during the current circumstances. However, we recommend that these surveys nevertheless move forward without delay. Here we explain why and how to do so. Although our advice is specific to institutions of higher education, it would apply just as well to nonacademic organizations wanting to understand member experiences of sexual harassment. While climate surveys can cover a number of topics (e.g., discrimination, safety, bullying, etc.), our focus here is on climate surveys that examine sexual harassment. Consistent with the 2018 NASEM report, we use the term “sexual harassment” in its broadest sense, referring to behaviors that derogate, humiliate, or violate people because of their sex or gender. These behaviors include many forms of wrongdoing, from gender-based insults to pornographic displays to unwanted sexual pursuit to sexual assault and rape (5).

## Why Not Postpone Surveys?

Some might wonder whether educational institutions ought to pause sexual harassment assessment efforts until social distancing ends and campuses return to normal. However, many institutions won’t resume full in-person instruction anytime soon. Distance learning and hybrid educational models (a combination of distance plus live instruction) will be with us for some time to come (6). Many campuses will continue to encourage—even mandate—working and schooling from home. Moreover, students remain enrolled in our institutions, some residing on or near campus. Staff and faculty remain employed, and some perform vital functions that must take place in person (e.g., custodians, maintenance personnel, food service workers, and healthcare providers). Sooner rather than later, we are going to need baseline assessments to understand whether and how harassment is happening in this “new normal” of higher education, including how sexual harassment takes on new forms in virtual environments.

Although the coronavirus has distanced many of us from one another, it has not stopped sex-based harassment and violence (or other harmful behaviors such as acts of racism or anti-LGBTQ bullying). Gender-harassing comments and gestures easily continue in electronic forums. In fact, technology-mediated misconduct may be on the rise. Considerably more work now takes place via digital media: video and audio calls, virtual chatrooms, learning management systems (e.g., Canvas and Blackboard), and social learning platforms (e.g., Yellowdig Engage and Perusall). According to research on cyber harassment, some people feel freer to behave badly when communicating through technology, especially when they can mask their identities (7, 8). Many students, staff, and faculty have already been the victims of “zoombombing,” in which online meetings and classes are disrupted by intrusions of sexist, racist, misogynistic, or pornographic content (9). Harassment even happens over email, where tone is difficult to convey [including student harassment of faculty and staff, known as “contrapower” harassment (10)].

Exacerbating these problems are the tensions running high in many families, due to COVID-related illness, isolation, unemployment, financial strain, and childcare demands (11, 12). In some homes, physical violence is becoming more frequent and more severe (13). LGBTQ+ students may be returning to homes that are unsupportive and unsafe (14). All of this means that home-based work or school environments have suddenly become more hostile, and possibly more dangerous, for many students, faculty, and staff in academia.

Higher education is shifting as a context, and so is sexual harassment. Harassment prevention and response efforts should shift as well, adapting to meet the needs of our communities in this new reality. To inform those efforts, we need up-to-date data on the forms that harassment takes and the factors that drive it. Regular climate surveys are critical for establishing baseline prevalence estimates of sexual harassment across institutions of higher education.

**Fig. 1.**
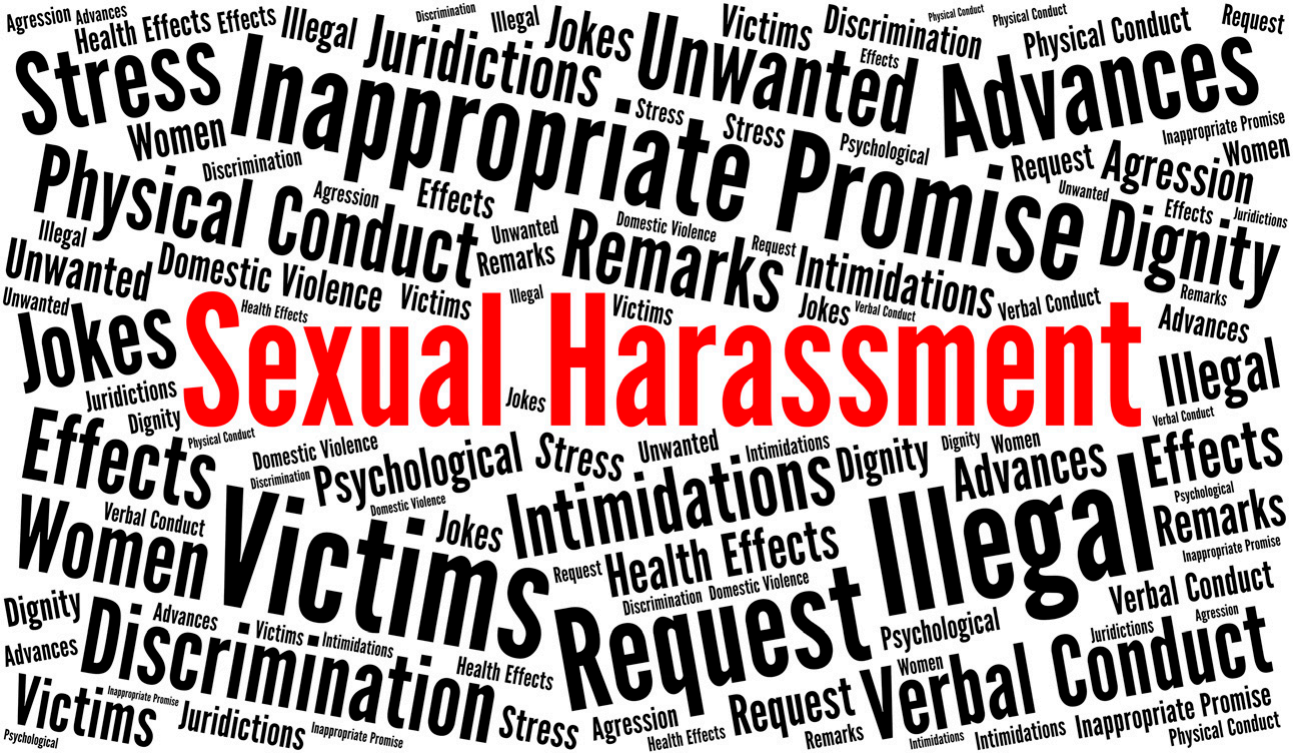
Word cloud of terms that describe types of sexual harassment, its impacts, and the legal implications. Image credit: Shutterstock/ricochet64.

A final reason for these surveys is that sexual harassment interferes with the core research and education missions of our institutions. Dozens of studies [summarized in the National Academies report (2)] have shown that sexual harassment, even when perpetrated online, derails work and well-being (Fig. 1). Creativity, concentration, and performance suffer. Many harassed faculty and staff leave for jobs elsewhere, resulting in a costly loss of talent to their institutions. The picture looks just as bleak for students: Their education takes a hit as they take measures to avoid hostile learning environments (e.g., changing advisors, changing majors, or dropping out of school). Such negative outcomes are likely exacerbated by the increased distractions inherent in working and learning from home and increased efforts needed to make sense of interpersonal interactions in remote environments. Climate survey efforts can reassure employees and students that their well-being remains important to their campus leaders. Resulting data can help institutions devise better strategies to combat sexual harassment (and other forms of harassment and discrimination) and curtail the harms left in its wake.

## How Best to Proceed?

For the reasons outlined above, sexual harassment surveys should continue in higher education, even during a pandemic. Surveys should follow the best practices reviewed in chapter 2 of the National Academies report (2). Doing so includes the use of scientifically informed tools to assess sexual harassment and its sequelae, such as those found in the Administrator Researcher Campus Climate Collaborative (ARC3) Survey [a free survey that includes the best-validated instruments available for assessing sexual harassment and violence (15–17)]. Several adjustments are in order, though, to ensure reasonable response rates and high-quality data.

First, leaders should take care to communicate with students, staff, and faculty with compassion; acknowledge that they are asking people to participate in a climate assessment during a difficult time; and emphasize that campus safety and equity remain top priorities.

Second, the response time requested of community members should be flexible. Surveys could offer options of immediate versus deferred participation. For instance, when surveying students, the following text could be used in survey invitations:

The COVID-19 pandemic impacts our entire campus community. It is important to continue our focus on student safety in all areas; although, we understand that now may or may not be a good time for you to complete this climate survey, and you may need flexibility during these uncertain times. Therefore, once you login to the survey website, you will have the option to either complete the climate survey immediately or defer the survey. If you choose to defer, we will email you an invitation again after 30 days to make sure you have the opportunity to complete the climate survey.*

Third, climate surveys should include questions about cyber sexual harassment (of any type: gender harassment, unwanted sexual attention, or sexual coercion). As noted, the move to remote work and teaching is spawning new manifestations of harassment based on gender, sex, race, and other social identities. New questions may be needed to capture these phenomena.

Fourth, the reference period of the assessment should be clear. A good sexual harassment survey asks students, staff, or faculty only about experiences from the *recent* past (past year or two), to avoid recall bias (2). In addition, consider asking people to report on experiences both before and after the switch to remote work/learning. Then, the institution can parse apart information about the traditional (in-person) climate versus the new (remote or hybrid) environment.

Finally, institutions might choose to include additional assessments specific to COVID-19 that would not normally appear on a sexual harassment survey. The NIH Office of Behavioral and Social Sciences Research (OBSSR) has compiled a comprehensive list of survey instruments that are being used to understand the impact of COVID-19 on people’s lives (18).

## Continuing the Fight

In many ways, the sudden shift to distance learning and telework heightens the urgency of surveys on sexual harassment (and other forms of interpersonal mistreatment). COVID-19 has made our educational and employment lives more complicated, stretching beyond the traditional confines of the academy. However, this does not lessen institutional responsibilities meant to ensure a safe and equitable environment for all students, staff, and faculty. We can’t check our civil rights obligations at the door when a public health crisis erupts. Sexual harassment in all forms—physical, verbal, or virtual—remains altogether impermissible.

We urge institutions of higher education to continue efforts to address sexual harassment, including climate surveys that assess and track it over time. If anything, this is more critical now that harassment is shape-shifting: moving online, emerging in new forms, and finding new ways to impair our lives.

Don’t let COVID disrupt your campus climate surveys, but do take time to adapt them to fit our strange new reality.

## Footnotes

*We are grateful to Kevin Swartout, Associate Professor of Psychology at Georgia State University, for suggesting this survey invitation language.

## References

[1] National Academies of Sciences, Engineering, and Medicine, Action Collaborative on Preventing Sexual Harassment in Higher Education. https://www.nationalacademies.org/our-work/action-collaborative-on-preventing-sexual-harassment-in-higher-education. Accessed 13 August 2020.

[2] National Academies of Sciences, Engineering, and Medicine, Sexual Harassment of Women: Climate, Culture, and Consequences in Academic Science, Engineering, and Medicine (National Academies Press, 2018). 29894119

[3] National Academy of Medicine, Action Collaborative on Clinician Well-Being and Resilience. https://nam.edu/initiatives/clinician-resilience-and-well-being/. Accessed 13 August 2020.

[4] National Academy of Medicine, Countering the U.S. Opioid Epidemic. https://nam.edu/programs/action-collaborative-on-countering-the-u-s-opioid-epidemic/. Accessed 13 August 2020.

[5] Berdahl J. L., Harassment based on sex: Protecting social status in the context of gender hierarchy. Acad. Manage. Rev. 32, 641–658 (2007).

[6] McMurtrie B., How the pandemic will change teaching on campus: The things that make learning effective in person need to be reimagined. *Chronicle of Higher Education*, 3 June 2020.

[7] Lim V. K. G., Teo T. S. H., Mind your e-manners: Impact of cyber incivility on employees’ work attitudes and behavior. Inf. Manage. 46, 419–425 (2009).

[8] Pearson C. M., Porath C. L., On the nature, consequences and remedies of workplace incivility: No time for “nice”? Think again. Acad. Manage. Exec. 19, 7–18 (2005).

[9] Redden E., ‘Zoombombers’ disrupt online classes with racist, pornographic content. *Inside Higher Ed*, 14 May 2020.

[10] DeSouza E. R., Frequency rates and correlates of contrapower harassment in higher education. J. Interpers. Violence 26, 158–188 (2011). 20448231 10.1177/0886260510362878

[11] Bennett J., ‘I feel like I have five jobs’: Moms navigate the pandemic. *NY Times*, 20 March 2020.

[12] Schmidt G., A federal agency studies additional benefits. *NY Times*, 4 June 2020. https://www.nytimes.com/2020/06/04/business/unemployment-jobless-claims-coronavirus.html. Accessed 13 August 2020.

[13] Taub A., A new Covid-19 crisis: Domestic abuse rises worldwide. *Intepreter*, 14 May 2020.

[14] Brown S., Covid-19 sent LGBTQ students back to unsupportive homes. That raises the risk they won’t return. *Chronicle of Higher Education*, 24 April 2020.

[15] Swartout K. M., Measuring campus sexual misconduct and its context: The Administrator-Researcher Campus Climate Consortium (ARC3) survey. Psychol. Trauma 11, 495–504 (2019). 30080069 10.1037/tra0000395

[16] Kingkade T., A supergroup of academics is trying to stop people who profit from campus rape. *HuffPost*, 2 May 2020.

[17] Tilley D. S., Wang W., Kolodetsky A., Yeatts P., Factor Analysis of the Administrator-Research Campus Climate Collaborative (ARC3) Survey. Health Educ. Behav. 47 (suppl. 1), 54S–69S (2020). 32452254 10.1177/1090198120911613

[18] National Institutes of Health Office of Behavioral and Social Sciences Research, Data from “COVID-19 OBSSR Research Tools.” https://www.nlm.nih.gov/dr2/COVID-19_BSSR_Research_Tools.pdf. Accessed 13 August 2020.

